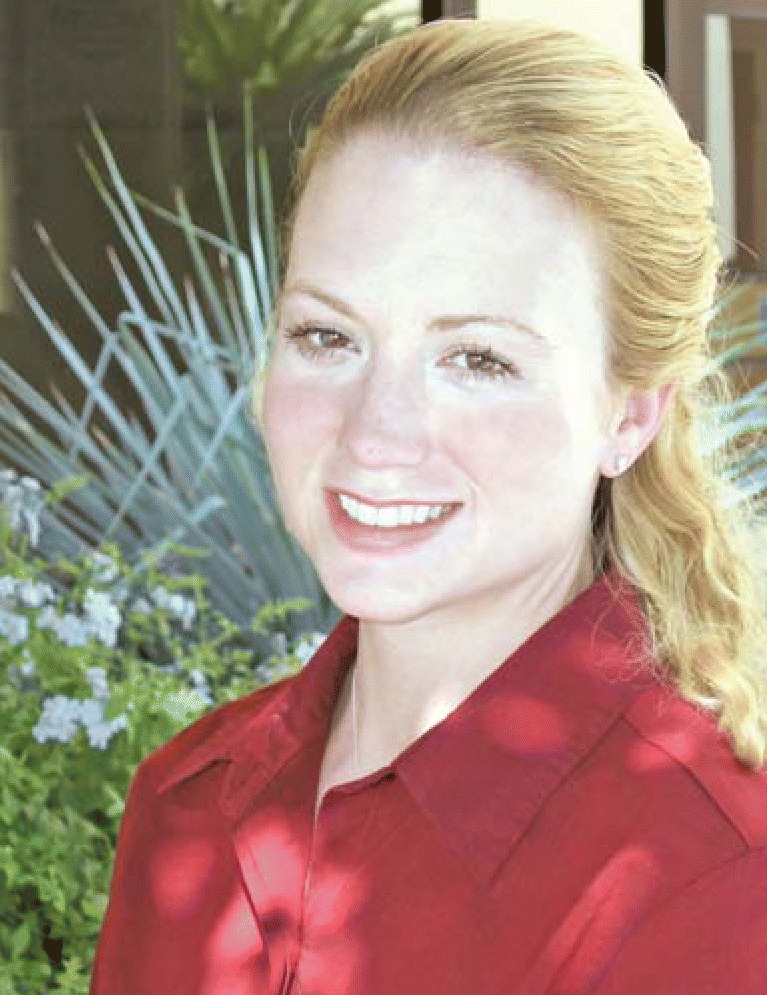# Tiffany G. Bredfeldt, University of Arizona: Recipient of the 2005 Karen Wetterhahn Memorial Award

**Published:** 2006-01

The Superfund Basic Research Program (SBRP) is pleased to announce that Ms. Tiffany G. Bredfeldt of the University of Arizona is the recipient of the eighth annual Karen Wetterhahn Memorial Award. The award will presented to Ms. Bredfeldt on 13 January 2006 at the SBRP annual meeting in New York, New York.

The SBRP presents this annual award to an outstanding scholar to pay tribute to the life and scientific accomplishments of Karen E. Wetterhahn, former director of the SBRP at Dartmouth College. Dr. Wetterhahn died in 1997 as the result of an accidental exposure to dimethylmercury. An acknowledged international expert on the effects of heavy metals on biologic systems, Dr. Wetterhahn was a leader in conducting research on how metals initiate cancer and other metal-induced human diseases at the molecular level. She fostered links among biology, chemistry, environmental studies, engineering, and medical science, insisting that “the life sciences are interdisciplinary.”

Ms. Bredfeldt is a magna cum laude graduate of the University of Arkansas, where she earned a B.S. in microbiology and minored in Spanish. She is in the fifth year of a Ph.D. program at the University of Arizona, where, under the guidance of Dr. A. Jay Gandolfi, she is working to identify which species of arsenic have the potential to malignantly transform human cells. In research that Dr. Gandolfi characterizes as “breakthrough,” Ms. Bredfeldt demonstrated that *a*) at environmentally relevant levels of arsenic exposure, human bladder cells can generate monomethylarsonous acid (MMAIII), an arsenic metabolite that is 20 times more toxic than inorganic arsenic, and *b*) MMAIII can transform human bladder cells into a new cancer cell line. This appears to be the first observation of MMAIII-induced cellular transformation of any human cell line—a truly exciting finding. Ms. Bredfeldt’s observations strongly support the notion that arsenic metabolites may be functioning as the ultimate toxicants in arsenic-induced pathologies due to their heightened toxicity compared with inorganic arsenic.

The NIEHS congratulates Ms. Bredfeldt on her research accomplishments and wishes her continued success in her scientific career.

## Figures and Tables

**Figure:**